# The antimicrobial resistance crisis: management through gene monitoring

**DOI:** 10.1098/rsob.160236

**Published:** 2016-11-09

**Authors:** Carolyn A. Michael, Ashley E. Franks, Maurizio Labbate

**Affiliations:** 1 School of Life Sciences, University of Technology Sydney, Sydney 2007, Australia; 2 ithree institute, University of Technology Sydney, Sydney 2007, Australia; 3 Department of Physiology, Anatomy and Microbiology, School of Life Sciences, La Trobe University, Melbourne, Victoria, Australia

**Keywords:** antimicrobial resistance, horizontal gene transfer, evolution, crisis management

## Abstract

Antimicrobial resistance (AMR) is an acknowledged crisis for humanity. Its genetic origins and dire potential outcomes are increasingly well understood. However, diagnostic techniques for monitoring the crisis are currently largely limited to enumerating the increasing incidence of resistant pathogens. Being the end-stage of the evolutionary process that produces antimicrobial resistant pathogens, these measurements, while diagnostic, are not prognostic, and so are not optimal in managing this crisis. A better test is required. Here, using insights from an understanding of evolutionary processes ruling the changing abundance of genes under selective pressure, we suggest a predictive framework for the AMR crisis. We then discuss the likely progression of resistance for both existing and prospective antimicrobial therapies. Finally, we suggest that by the environmental monitoring of resistance gene frequency, resistance may be detected and tracked presumptively, and how this tool may be used to guide decision-making in the local and global use of antimicrobials.

## 1. Introduction

Antimicrobials in general and antibiotics specifically have had a large beneficial impact on humanity by reducing or preventing infection, disease and epidemic, and also through their improvement of food production and security. However, the number of infectious diseases whose treatment is complicated or even precluded by the failure of existing antimicrobial therapies continues to increase and is already a significant crisis for humanity [1–3]. Consequently, the prospect of the failure of antimicrobials as a class and the resulting impact on humanity, particularly in view of our rapidly enlarging and increasingly connected population, is dire [3].

It is widely accepted that the antimicrobial resistance (AMR) crisis has been caused by the widespread and indiscriminate use of antimicrobials both clinically and in the environment [4,5]. This intense usage has caused those characters that confer resistance to antimicrobials in microorganisms to become both more common and diverse in microbial communities globally. Resistance to antibiotics, in particular, has increased globally since the widespread introduction of penicillin during World War II. In this time, widespread resistance to not only penicillin, but also its derivatives and all other available antibiotics, has become evident.

To date, the management of this crisis, while multifactorial and widely acknowledged as necessary, has failed to halt or even meaningfully decrease the incidence of AMR in previously manageable diseases [1,2]. A great deal of attention has been applied to the need for development of novel antimicrobials and therapies, public and clinical education, as well as legislative reform [1,2]. Yet little focus has been given to the problem of rapidly and accurately monitoring the root cause of the AMR crisis. In the clinical world, accurate and presumptive diagnosis is a cornerstone of modern medicine. Being able to sensitively monitor the progress of disease and its treatment enables swift, accurate and decisive action to be taken to preserve the health of the patient. In measuring the progress of the AMR crisis, the practice of counting the increasing variety of resistant pathogens in clinical environments [2] only documents the last stage of the process whereby resistance arises, proliferates, becomes associated with a pathogenic organism and ultimately comes to the attention of humanity as a newly resistant disease. Clearly, a more presumptive diagnostic methodology is required.

The individual genetic mechanisms whereby genetic material is modified and mobilized are becoming well understood [5,6]. It is known that DNA, often containing many types of adaptive gene, including AMR, is routinely transferred among diverse types of microbes [7]. So, organisms that are initially sensitive to one or more antimicrobial can acquire existing AMR genes and so become resistant. Importantly, this resistance may then be heritable. We now recognize also that AMR phenotypes and the genes that cause them are distinct from pathogenicity, and so evolve and are selected for separately. So, the development or acquisition of AMR is not restricted to pathogenic organisms. Rather, AMR can be acquired broadly in the microbial world and will proliferate when even a low level of antimicrobial stress is applied. By only measuring in clinical environments the incidence of pathogenic organisms that have acquired resistance genes, the huge environmental reservoir of resistance genes available to pathogens is not considered. By contrast, by directly monitoring the entire genetic resistance resource available to microbes, better estimates of the changing scope of the AMR problem can be made. Accordingly, we recommend that the frequency of resistance providing genes in both clinical and in non-clinical environments be measured and their changing frequency analysed over time. This apparently difficult task is made substantially simpler because AMR phenotypes are often provided by families of single genes such as the *bla, aadA, aadB* and *sul* types of gene, as well as others, so making the detection and subsequent quantification of this subset of resistance genes in complex mixtures relatively simple [8]. Then, with appropriate sampling strategies, both spatial and longitudinal data of AMR gene frequency can be analysed in the light of our understanding of the evolutionary process, to track the historic progress of resistance genes and then predict their rise and fall in microbial communities.

While it is common to assume that evolution proceeds over geological time, there are also numerous examples of the rapid evolution and consequent rise of organisms containing novel traits. Perhaps the most graphic of these is the rapid and widespread advent of AMR itself. Humans, in applying a wide range of antimicrobials in high doses over many years, have caused a strong, widespread and persistent selective pressure to be applied to the microbial world. This selective pressure has resulted in a rapid evolutionary response [9–12]. This response has seen the spread of resistance genes previously sequestered in low numbers within microbial communities, as well as the production of new AMR-providing genes. These resistance genes, in providing an advantage under antimicrobial stress, enable the survival of their hosts, and so become more common and may even become ubiquitous in the evolving microbial community.

The ability to produce novel genes demonstrates that the microbial world is remarkably protean in producing new ways of combatting or otherwise taking advantage of both existing and even completely synthetic challenges [13,14]. Therefore, it is also likely that there will be no single or even durable solution in the future by which microbial infections may be controlled. Consequently, in the absence of such a serendipitous solution to the AMR problem, the measurement and subsequent management of the processes whereby AMR arises offer the best hope for long-term control.

Existing environmental studies of the presence of AMR genes show that resistance genes are widely distributed and can even be found in environments that are not apparently subject to antimicrobial stress, albeit at low frequencies [12,15]. The application of even low levels of antimicrobial in even such relatively isolated environments can produce genetic changes that ultimately select for resistance genes [16]. These studies imply that in order for environmental reservoirs of resistance genes to be reduced to or maintained at low levels, selective pressures that conserve these particular genes must be largely eliminated in those environments. To prevent pollution with resistance genes, migration between highly and less selective pressure environments should also be controlled. The complete withdrawal of an antibiotic from an environment, particularly in a clinical context, may appear to be a drastic and possibly futile measure; however, evolutionary models of gene reduction in the absence of selection show encouraging results [17]. Similarly, in studies of the incidence of phenotypic resistance in environments where the causative antibiotic was either removed or no longer required due to intense vaccination programmes, a significant reduction in the rate of phenotypic resistance was seen in only a few years [18–20]. While not elucidating the underlying gene frequencies, these results suggest that effective measures exist which, when coupled with gene frequency monitoring, can manage the AMR crisis.

In order to substantiate our recommendation, we now consider the rise of AMR from an evolutionary perspective.

## 2. Measuring evolution

The adaptive abilities of individual organisms arise from their genetic constitution (genome) and its differential expression. Therefore, changes in the genome of an individual can result in changes in their adaptive abilities. These changes may arise through the addition or creation of novel genetic material, through the changing modulation of the expression of existing genes or else through the removal of genetic material.

### 2.1. Adaptation through the addition of genetic material to microbial genomes

New phenotypes can arise through the addition of new genetic material. This addition may be as a result of mutation, whereby new genes are created de novo from previously non-coding DNA or else from modification of a copy of an existing functional gene [21,22]. Additionally, mutation through acting upon the regulatory areas of DNA associated with an existing gene may alter its expression and so also provide an adaptive phenotype through this change in regulation. An example of this is the upregulation of membrane efflux pumps in *Mycobacterium tuberculosis*, so enabling the organism to more rapidly remove antibiotic from the cell [23]. The rate at which mutation proceeds is generally thought to be very slow, with estimates ranging from 1 DNA base pair change in 10^4^ to 1 in 10^9^ per generation. However, under duress, microbial cells may facilitate mutation under a generalized SOS response, increasing to many base pair changes per genome per generation. In considering that the average gene may be more than a kilobase long and that the process of changing one functional gene into another involves many random base changes, it is clear that in the absence of instances of hyper-mutation, the production of novel genes may be both a long and serendipitous process. While the average rate of mutation may be low, continued strong selective pressure across a wide range of environments means that even rare resistance providing mutations will be widely available to be selected for. So, effectively monitoring resistance genes will also always require screening for such novel genes that can arise at any time.

Existing genetic material, but novel to the host cell, may also be introduced by the various processes of horizontal gene transfer (HGT) [24]. These processes transfer DNA and whatever genes it may contain from one cell to another, largely irrespective of their differing lineages. Horizontally transferrable DNA includes a vast library of adaptive characteristics distributed across all microbial communities and stored over geological periods of time [10,11]. HGT has been shown to be largely responsible for the acquisition by sensitive microbes of single and multiple AMR-determining genes, and so is a major contributor to the current AMR crisis [8,25]. It is known that many of the individual processes contributing to HGT are themselves responsive to environmental stress. For example, the integrase gene that causes the excision or insertion of mobile gene cassettes within integron structures is upregulated under cellular stress, thus increasing the rate at which gene-containing cassettes are mobilized [26,27]. Similarly, the natural transformation process of many microbes, allowing the uptake of DNA from the environment, is increased under environmental stress [28]. However, while the overall rate of HGT may increase under stress, even its basal rate continually enables the distribution of novel phenotypes across the microbial world.

The processes whereby new phenotypes are produced act at variable rates. Both the HGT and mutational responses have been shown to provide adaptive phenotypes to microbial strains within hours of the onset of an intense stressor [29] (A. Robinson 2016, personal communication). However, despite the ability of microbial communities to produce a more rapid adaptive response under duress, even under negligible stress such new phenotypes are still produced within microbial communities [16]. Therefore, the determining factor in predicting whether these genes and their associated phenotypes become common in the environment is the selective process rather than the rate at which they are generated. Additionally, where an adaptive gene comes to predominate within a microbial community, its frequency in adjacent communities will increase through microbial migration. Even distant environments will eventually receive migratory genes by this process. This phenomenon is graphically illustrated by the presence of AMR genes, particularly those associated with promiscuous class 1 integrons being found in environments not apparently under antimicrobial selective pressure, such as Scandinavian lake bottoms and desert soils [10,11,30]. This further underscores the importance of selection rather than the genetic origin of genes in determining the abundance of AMR.

**Figure 1.**
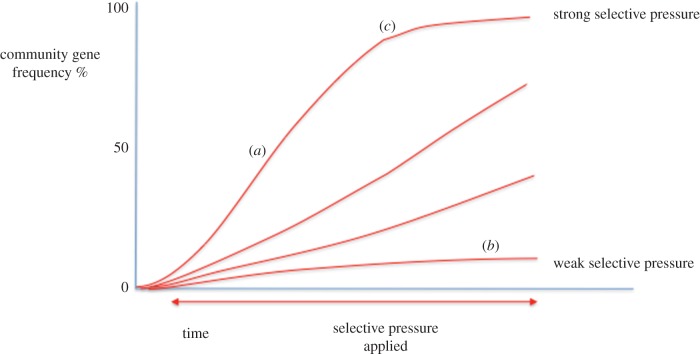
Change in community gene frequency under selective pressure. The rate at which gene frequency rises within a microbial community is largely dependent upon the strength of the selective pressure. Where a selective pressure significantly impairs the reproductive success of a large proportion of the community, gene frequency rises rapidly even though overall community numbers may decrease (*a*). Conversely, where the impairment is less severe or else does not affect a large proportion of the community, this leads to a lower rate of increase (*b*). This latter effect, where the proportion of the population affected by a stressor decreases as the gene becomes more frequent, leads to gene frequency asymptotically approaching fixation (*c*).

Once a new functional gene and its corresponding phenotype arises, selection by ambient conditions will determine its frequency within a microbial community. In simple terms, under a single strong selective pressure, an adaptive gene that provides advantage to its host will increase in frequency within the community over relatively few generations and shortly thereafter may be ubiquitous. The rate at which the gene frequency of a selected for gene increases over time, while showing a characteristic shape when graphed (figure 1), is nevertheless dependent upon many factors [31]. These factors include the strength of the selective pressure, the fitness of the population under pressure as well as the presence of other selective pressures acting within the environment. Additionally, the maximum frequency attained by a gene within a community will be influenced by competition with other genes supplying similar resistance phenotypes and also the degree to which a gene may be easily mobilized. Nevertheless, by knowing the frequency of a particular adaptive gene within an environment, combined with its varying rate of change over time, an estimate of the community's current location on the curve can be made. Consequently, predictions can be made regarding the gene's ultimate frequency within the microbial community and also how long that might take [32].

### 2.2. Adaptation through genome reduction

While the addition of new genetic material is often responsible for the provision of novel adaptive phenotypes, there are also inevitable costs to this addition, so decreasing the fitness of the host. These costs include the replicative and expression burden imposed by the presence of new genes as well as the possibility of pleiotropic/epistatic interference of imported genes with existing biochemical pathways [33]. It must be noted that in some cases the addition of adaptive genes can also increase the fitness of an organism even where selective pressure for the apparent phenotype provided by the gene is absent [34]. In general, where genes and the phenotypes they provide do not actively contribute to the fitness of their host organism, they tend to be inactivated or lost.

A variety of processes contribute to genome reduction. Perhaps the most pervasive is the accumulation of random mutations combined with the observed bias towards base pair deletions [35]. This process, when acting upon DNA that is not continually selected for, will slowly modify and erode the sequence, first causing genes to lose their function, and culminating in their loss to the genome. So, with no other processes at work, this slow erosion results in a decrease in adaptive gene frequency within a population.

Where an organism or community, initially under selective pressure, enters a largely benign environment, the advantage conferred by adaptive genes is lost, and often results in the rapid loss of large segments of DNA from individual organisms. Recent work has shown losses of many kb in single and multiple indels when virulence and resistance factors contained on mobile genetic elements are lost through the processes of HGT [36,37]. At a further extreme, organisms entering a nutrient-poor or single-nutrient environment have been seen to rapidly delete even core genetic mechanisms associated with metabolism of alternate nutrients [37]. These effects are most often seen in endosymbionts, where in later stages not only nutritive genes but also those associated with replication (as well as other core functions) are lost. Examples of this process include the markedly diminished genome of *M. leprae* when compared with its close relative *M. tuberculosis* and many others [23].

**Figure 2.**
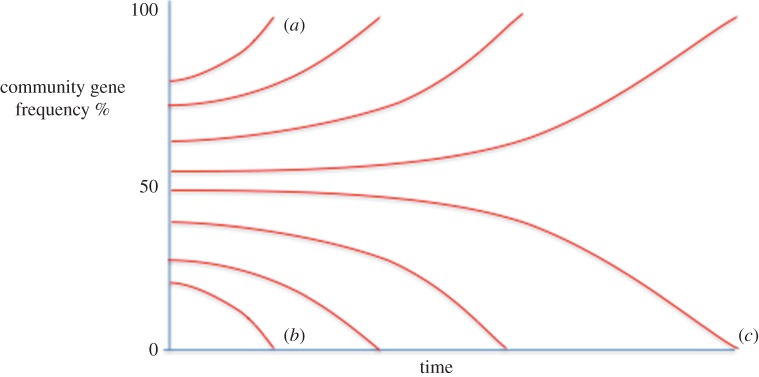
Change of community gene frequency due to HGT and particularly natural transformation and recombination. The processes of HGT are stochastic. So, in the absence of selection to drive an increase in gene frequency the chance of an existing gene being excised from the host DNA and subsequently lost is the same as the gene frequency across the contributing community. Accordingly, where a gene is common in the community (*a*) it will become more so. Similarly, where a gene is rare (*b*), it will rapidly be lost from the community. The rate at which genes are lost or gained is also proportional to their frequency (*c*).

Finally, the process of natural transformation has been shown to be much more widespread and effective than previously thought [28,38,39]. This process, whereby naked DNA is taken up from the environment, enables the rapid and frequent uptake of large (many kb) DNA fragments. Where such DNA fragments themselves contain mobile DNA such as transposons or gene cassettes, such elements may then be integrated into the host DNA through mechanisms of HGT such as transposition or site-specific recombination. However, where the introduced DNA does not include a mobile element, homologous recombination alone may result in the excision of a segment of host DNA containing a resident mobile element so eliminating it from the genome and from subsequent replication. The frequency with which such eliminations of mobile elements occur is inversely proportional to the frequency of such elements within the microbial community. Thus, where a mobile element is rare within a community, in the absence of selection to increase its frequency, it will be rapidly lost. Similarly, where a mobile element is common or even predominates within a community, the process of homologous recombination following transformation will serve to maintain or even increase its frequency (figure 2).

### 2.3. Spatial considerations

The foregoing discussion assumes that the community undergoing selection is isolated from all other microbial communities. In practice, migration between microbial communities must also be considered in modelling the ultimate evolutionary fate of genes [40,41].

**Figure 3.**
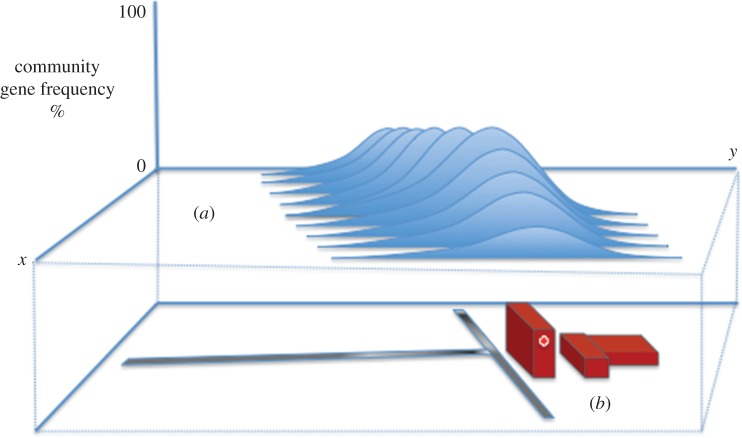
A hypothetical example of the changing geographical distribution of resistance genes. The changing spatial distribution of resistance genes can be used as an indicative measure of gene migration and hence the effectiveness of both stewardship and control programmes. Additionally, the availability of areas of low gene frequencies adjacent to areas of intense antimicrobial usage (*a*) may offer the ability to ‘dilute’ areas of high resistance gene frequency and so hasten the removal of resistance genes from the community. By applying geographical information system (GIS) or network analysis approaches to such information, correlations with environmental and socioeconomic variables may be drawn (*b*).

Where selection occurs in a single location, as would be the case in a hospital utilizing a novel antimicrobial, selection for resistance factors either present or imported into the hospital would increase their gene frequency in that location. With the introduction of organisms from outside the hospital environment not containing resistance genes, the overall rise of resistance gene frequency within the hospital is delayed as the resistance gene is diluted within the hospital's microbial community. Conversely, the migration of resistance-gene-carrying organisms from the selective hospital environment to the surrounding non-selective environment creates a wider reservoir of selective genes that may be reintroduced into the hospital environment after selection there has ceased, so delaying the reduction of resistance gene frequency within the hospital. This effect would become more pronounced if a selective pressure, even at a low level, was applied to the environment surrounding the hospital, thus delaying or preventing the resistance genes from being eliminated there (figure 3).

### 2.4. Summation

**Figure 4.**
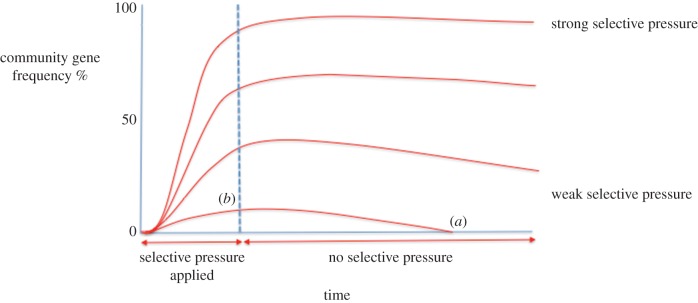
The overall change in community gene frequency with time. When the mechanisms that compete to change gene frequency within a community are considered together, it is clear that in order to rapidly reduce gene frequency (*a*), a low starting frequency (*b*) in the absence of selection is required. One way this may be achieved is to apply only a weak selective pressure (*b*) to the environment. Alternatively, early intervention to remove high selective pressures before a high gene frequency is achieved may be effective.

When considered as a complete process (figure 4), that is, a community undergoing selection followed by the withdrawal of selection, it is clear that the ultimate fate of an adaptive gene under selection within the community is dependent on the strength of the selective pressure applied and the duration for which it is applied. A strong selective pressure applied for long periods of time will increase the gene frequency of an adaptive gene to the point where its removal from the community metagenome after the removal of the stressor will take many generations, if it happens at all. Conversely, where a selective pressure is applied weakly, locally and for short durations, the frequency of adaptive genes will not rise to high levels. Therefore, upon the removal of the stressor upon such communities, the frequency of such genes would be rapidly decreased in the metagenome. Consequently, this information when applied to resistance genes, with knowledge of the history of resistance gene frequency within an environment, provides both diagnostic and prognostic information that can guide the subsequent application of environmental stressors such as antimicrobials.

## 3. Implementation

We recommend that, in conjunction with the use of antimicrobials, a programme for monitoring the complementary resistance gene frequencies in environments within and surrounding the antimicrobial usage sites should be implemented.

### 3.1. Sampling

Sampling the environmental metagenome requires that sampling be both reproducible and repeatable over the life of the experiment. This is especially important in an ongoing programme of monitoring resistance gene frequencies in environments including human society. The principal interest of such a diagnostic study is to monitor resistance genes with a particular interest in both their presence and availability to potential human pathogens. Thus, appropriate sampling techniques necessarily invoke a bias towards sampling the microbial metagenome in close proximity to large numbers of humans.

It is current practice in many hospitals to sample the clinical environment for the presence of resistant pathogens. Most often, sampling consists of surface swabs and air samples taken from locations within the hospital. These samples are variously collected onto swabs or membrane filters that are subsequently cultured in selective media to identify both pathogens and their resistance profiles. We recommend that this procedure continue so as to identify newly arisen resistance genes not currently screened for. In addition to these culturable samples, we recommend that parallel swab and filter samples for direct metagenomic DNA extraction be taken. Direct extraction of DNA samples without intervening culture will capture the DNA of both cultural and unculturable microorganisms in the environment and retain the relative abundances of both organisms and the genes they contain [42].

In addition to clinical environments that are presumed to be a focus of resistance gene prevalence, comparative sampling of surrounding environments frequented by humans but not exposed to high antimicrobial usage should also be undertaken. Taking surface swabs and air samples both for culture and DNA analysis in the undercover areas of shopping precincts or transport hubs at increasing ranges from the clinical environment would be appropriate.

While necessarily only accessing that fraction of the microbiome that survives transit through the human gut, samples taken from sewerage may be a useful adjunct. Such samples taken from sewerage mains draining urban and clinical environments may be more reproducible and less obtrusive to the general public, and so may offer an alternate means of implementing a gene frequency monitoring programme.

### 3.2. Sampling frequencies

Because quantitative results are required from the proposed work, it is necessary to quantify the variability inherent in the sampling and analysis techniques. So, environmental samples should always be collected in replicates. Periodically, replicate studies of each stage of the analytical process should be undertaken to establish the reproducibility of the methods used. Quantitative studies of metagenomic DNA have shown that less than a 10% Standard Error is routinely achievable, with the PCR step being the most variable portion of the analytical routine [43].

Once the variability inherent in the methodology is known, the sampling period may be set when the difference in sample values taken at subsequent times exceeds the methodological variation.

### 3.3. Measuring gene frequency

A wide variety of existing molecular techniques have been demonstrated to recover and analyse environmental metagenomic DNA [15,16,44]. These techniques include next-generation sequencing as well as chip-based methodologies, though perhaps the simplest and most cost-effective methods use quantitative PCR (QPCR). Using established techniques to gather environmental DNA, resistance gene frequency may be simply established by the ratio of the QPCR value of the resistance gene or gene family, relative to generally conserved genes such as *16s rRNA* or *rpo*B [30,45,46]. Because resistance genes are subject to selection they are often seen to diversify into gene families such as the hundred or more different *bla* genes. These gene families can be usefully measured with a limited number of QPCRs through the use of either degenerate or consensus PCR primers. Genes providing AMR phenotypes not provided by known genes can be defined through continued phenotypic testing and subsequent DNA sequencing.

Where resistance genes are known to be less mobile, as in the case of mutated genes or upregulated biochemical pathways, rather than measuring the total microbiome, a strain-specifically conserved reference gene may be utilized instead of *rpo*B.

The gene frequency information gathered longitudinally in the real world is unlikely to produce the smooth curves seen in the figures shown. Rather, sources of variability including variations in experimental technique, environmental changes as well as the influence of specific events such as epidemics or the introduction of vaccines will necessarily affect the results. Yet measuring and then accounting for such variability is already common in a variety of routine pathological methodologies and quality control programmes [47]. Consequently, measuring gene frequencies and then accounting for variability will allow overarching trends in resistance gene frequency to become evident.

### 3.4. Geopolitical considerations

It is perhaps beyond the purview of this work to suggest geopolitical structures whereby the AMR crisis can be managed. We note, however, that regional initiatives can produce short-term results beneficial to the local communities. The literature demonstrates that the frequency of resistance genes in a region can decrease markedly upon the withdrawal of selective pressure [17–19]. However, the AMR crisis is a global microbial evolutionary problem. The global ecology does not consist of an assemblage of discrete and isolated ecologies. Rather, the ecology of the planet is interconnected, and the increasingly large and mobile human population only facilitates this interconnection. Accordingly, the AMR crisis requires a global perspective in effectively managing it.

In human terms, the world is sub-divided into many distinct administrative units such as states and countries, each of suitable size for the implementation of initiatives to regionally manage AMR. However, the global nature of the problem suggests that global coordination of management initiatives is also required. There exist a number peak health bodies, such as the World Health Organization, the Centers for Disease Control and Prevention, and others, currently concerned by the AMR crisis whose advisory roles to country governments and global perspective would be appropriate in facilitating the implementation of the additional measures we suggest here. Further, it is hoped that our academic colleagues will also commence projects to locally longitudinally measure the changing frequency of resistance genes and in doing so refine the techniques involved and also offer insights into issues in the management of the crisis. We see that such efforts will form the basis of a broad-based and rigorous AMR management programme into the future.

## 4. Stewardship of existing antimicrobials

### 4.1. Monitoring the frequency of currently prevalent resistance genes

Trends in resistance gene frequency over time indicate a number of factors that are relevant to the management of AMR. By considering the rise or fall of gene frequency and at what rate, an estimate of the future prevalence of that gene can be made. For example, a low frequency of a particular resistance gene with an exponentially increasing trend indicates a strong current selective pressure for that gene. Similarly, a high-frequency gene without an increasing trend could indicate saturation of the environment with that gene.

By including other indicators, additional trends can be established. By continued monitoring of phenotypic resistance, as is already done in enumerating resistant pathogens, useful additional information is provided. For example, where increasing phenotypic resistance is seen despite a decreasing frequency of the monitored resistance gene, it could indicate the presence and increasing abundance of a novel resistance gene in the community. Further, an increase in resistance gene frequency without a corresponding rise in the incidence of resistant pathogens suggests a proliferation of the gene outside pathogenic hosts as the diversity and abundance of the genetic contexts for the resistance gene increase in the environment. While not of immediate concern clinically, this increased abundance of resistance genes in the metagenomic pool available to sensitive pathogens is of distinct concern in the ongoing management of AMR.

By including spatial variations in gene frequency measurements even more information would be gained. For example, a high resistance gene frequency localized to a clinical setting would indicate the effectiveness of containment measures, and would also suggest that the removal of selective pressure might produce a rapid dilution of resistance gene frequency through inward migration from surrounding communities. Conversely, slowly increasing gene frequencies in non-clinical environments could indicate that management measures were not entirely effective and, perhaps more importantly, that the reservoir of non-resistant strains in the larger environment was decreasing.

The stewardship of existing antimicrobials requires two broad areas of consideration in order both to ensure current utility and also to maintain long-term efficacy. These are the minimization of overall antimicrobial usage and the isolation of necessary antimicrobial usage from the wider microbial environment.

The first of these considerations requires that antimicrobials be administered in the lowest possible dose and for the shortest possible time required in order to achieve the desired clinical result. While lowered antimicrobial usage already forms part of the global strategy for managing AMR, by monitoring gene frequencies as a result of the use of antimicrobials, levels of usage that take into account AMR can be established. For example, where resistance genes specific to one type of antimicrobial are notably more frequent than genes against other antimicrobials, recommendations can be made that, where clinically appropriate, these alternative antimicrobials should be used preferentially.

The second consideration ideally requires that, particularly in a clinical context, an antimicrobial's metabolic breakdown products, as well as the microbial community exposed to its use, should be isolated from the wider microbial community. This means that resistance genes and the stressors that facilitate them will all be isolated from the general environment, thus reducing the opportunity for widespread dissemination of any resistance genes present. While apparently draconian in concept, the implementation of such measures is only slightly more complex than the currently accepted clinical techniques of isolation/quarantine wards and barrier nursing. The addition of PC2 laboratory-type precautions combined with measures to sterilize all effluvium from such facilities should prove effective in limiting both resistance gene and antimicrobial stressor flow to the environment. In the implementation of new antimicrobials and therapies where resistance gene frequency is low, such measures will ensure that treatment remains effective and hence rapid while preserving the long-term utility of the antimicrobial.

### 4.2. Stewardship of novel antimicrobials

An important strategy in managing the AMR crisis is the search for and development of new antimicrobial agents as well as better diagnostics and therapies [48–50]. Our existing antimicrobial repertoire has its origins in the microbial world, where these materials have been used in the long-term competition among differing types of microbe, and so it would seem likely that new antimicrobials might be found in the same arena. Similarly, resistance genes themselves have their origins deep within microbial contention [25]. So, it is likely that any new antimicrobial found will also have a suite of complementary resistance phenotypes already resident in the environment at some low frequency. In the implementation of new antimicrobials for the improvement human health, genes contributing to these resistance phenotypes should be identified before there is any widespread utilization of the new antimicrobial. The frequency of these resistance genes should subsequently be monitored both where the antimicrobial is given and in the surrounding environment, with any increase being seen as cause to consider withdrawing the antimicrobial or reducing its use until the corresponding resistance gene frequency has reduced. From the previous theoretical discussion, it is seen that the lower the gene frequency at which selective pressure is removed, the faster will a microbial community reduce or eliminate such genes. Consequently, in husbanding the long-term effectiveness of new antimicrobials, identifying resistance genes and then subsequently monitoring their frequency will enable objective decisions to be made in the use of these therapeutic materials for both the short-term benefit of patients as well as the long-term stewardship of the antimicrobial.

Of course, it is likely that novel forms of resistance will also become evident over time. Therefore, just as the presence of existing resistance genes were to be determined during the development of novel antimicrobials, continual surveillance for new resistance genes, as is currently the practice, will still be required.

## 5. Limitations

The suggested protocols discussed in this work are intended as an effective diagnostic technique for measuring the changing frequency of resistance genes in order to make decisions on the ongoing use of antimicrobials. It should be noted that diagnostic techniques are intended to measure a key parameter indicative of a disease to provide information for the timely management of that disease. This is distinct in both application and intent from research techniques that seek to elucidate the disease and all of its causes. We acknowledge that microbial ecology is a complex and as yet not completely defined discipline, and that many factors and processes (such as organismal stratification, genetic linkage of AMR genes and the changing rate of HGT) within microbial communities exist that may either enhance or impede the rate at which AMR genes either proliferate or decline. We also acknowledge that as a diagnostic technique, the measurement of AMR gene frequencies is not intended to elucidate these individual effects. Rather, the measurement of the changing incidence of AMR genes will demonstrate the result of the sum of these effects upon the microbial community and so will remain a useful diagnostic of the environmental AMR gene resource either present in or available to pathogens.

Nevertheless, the scientific resource represented by the stored metagenomic DNA samples collected initially for its diagnostic value may well be able to elucidate many research questions through the use of supplementary techniques such as targeted PCR or bulk sequencing. Consequently, while the initial intent of our proposed technique is diagnostic in nature, the potential for concurrent pure research is not neglected.

## 6. Discussion

By observing the gain or loss of adaptive genes from microbial communities over time, trends will become evident and estimates of the ultimate fate of such genes may be made. In considering genes conferring resistance to antimicrobials currently in use, it may well be that many resistance genes have already reached a high frequency in local and global communities, and so their decrease and subsequent loss from these environments is unlikely. However, as yet, work estimating the gene frequency of resistance genes across environments as well as longitudinally has not been done, and so the prediction of such a grim outcome is by no means certain. Indeed, the fact that sensitivity to even the oldest of the routinely used antibiotics (penicillin) remains detectable in clinical pathogens suggests that the frequency of resistance-causing genes is currently below saturation in the environments examined (data not shown). Similarly, while multiply and even completely resistant pathogens are being detected globally, they also remain relatively rare in most environments (data not shown). These gross observations suggest that the AMR crisis in the sampled environments has not yet reached an irrecoverable state. Nevertheless, by making measurements of resistance gene frequency directly, it will be possible to determine if these empirical observations in fact reflect an increasing, static or decreasing trend in the distribution and abundance of resistance genes. By measuring how these parameters vary over geographical distances across areas where the application of antimicrobials is occurring, an accurate view of the state of the evolutionary process will be gained, and this in turn will suggest measures to manage it and will also offer a means to judge the efficacy of these measures.

As an indicator of the utility of monitoring gene frequencies, it is useful to note that some studies have been completed which measure the abundance of either resistance genes or their DNA mobilizing hosts at a single point in time [15,30]. These studies have illustrated the presence of resistance genes in environments not directly associated with intense antimicrobial usage and the proclivity of these environments to select for these genes even under very low levels of applied antimicrobial stressors [15]. This information, while not indicating future trends, is nevertheless useful in demonstrating that resistance genes are widely spread, even though these studies do not elucidate whether their presence is due to recent arrival or else that they are ubiquitous and present at a low level in many environments. This work also shows that molecular methods to assess genetic constitution of environmental metagenomes is not only practical, but also sensitive and specific. Consequently, the routine longitudinal measurement of antibiotic resistance gene frequencies from environmental samples can easily become a regular feature in the management of the AMR crisis.

## 7. Conclusion

An uncontained AMR crisis promises an increased incidence and a wider variety of infectious diseases across the entire human population. Additionally, many medical and quasi-medical procedures, currently blithely undertaken, will incur increasing risk of infection and death. Less obvious consequences include threats to food security and increasing ramifications for the individual human microbiota, as well as the wider ecology. From this perspective, efforts to monitor, ameliorate and perhaps manage the AMR crisis, currently and into the foreseeable future become a reasonable endeavour. Consequently, our suggestions here of implementing techniques for monitoring resistance gene frequency, and hence managing the AMR crisis, rather than being an onerous imposition on the resources of the global community, become a necessary adjunct to human society.
